# Control of light emission of quantum emitters coupled to silicon nanoantenna using cylindrical vector beams

**DOI:** 10.1038/s41377-023-01229-9

**Published:** 2023-09-19

**Authors:** Martin Montagnac, Yoann Brûlé, Aurélien Cuche, Jean-Marie Poumirol, Sébastien J. Weber, Jonas Müller, Guilhem Larrieu, Vincent Larrey, Franck Fournel, Olivier Boisron, Bruno Masenelli, Gérard Colas des Francs, Gonzague Agez, Vincent Paillard

**Affiliations:** 1 CEMES-CNRS, Université de Toulouse, Toulouse, France; 2 ICB, Université de Bourgogne, CNRS, Dijon, France; 3 LAAS-CNRS, Université de Toulouse, Toulouse, France; 4 CEA-LETI, Université Grenoble-Alpes, Grenoble, France; 5 Université de Lyon, Université Lyon 1, CNRS UMR 5510, ILM, Villeurbanne, France; 6 Université de Lyon, INSA Lyon, CNRS, Ecole Centrale de Lyon, Université Lyon 1, CPE, UMR 5270, INL, Villeurbanne, France

**Keywords:** Nanophotonics and plasmonics, Sub-wavelength optics

## Abstract

Light emission of europium (Eu^3+^) ions placed in the vicinity of optically resonant nanoantennas is usually controlled by tailoring the local density of photon states (LDOS). We show that the polarization and shape of the excitation beam can also be used to manipulate light emission, as azimuthally or radially polarized cylindrical vector beam offers to spatially shape the electric and magnetic fields, in addition to the effect of silicon nanorings (Si-NRs) used as nanoantennas. The photoluminescence (PL) mappings of the Eu^3+^ transitions and the Si phonon mappings are strongly dependent of both the excitation beam and the Si-NR dimensions. The experimental results of Raman scattering and photoluminescence are confirmed by numerical simulations of the near-field intensity in the Si nanoantenna and in the Eu^3+^-doped film, respectively. The branching ratios obtained from the experimental PL maps also reveal a redistribution of the electric and magnetic emission channels. Our results show that it could be possible to spatially control both electric and magnetic dipolar emission of Eu^3+^ ions by switching the laser beam polarization, hence the near field at the excitation wavelength, and the electric and magnetic LDOS at the emission wavelength. This paves the way for optimized geometries taking advantage of both excitation and emission processes.

## Abstract (web summary)

Photoluminescence of Eu^3+^-doped thin film on a silicon nanoring. The photoluminescence enhancement around the resonant nanoantenna mimics the near-field hot spots tailored by radially or azimuthally polarized cylindrical vector beam.

## Graphical abstract

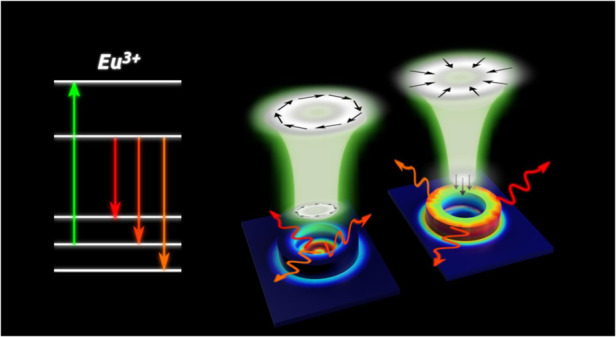

## Introduction

Enhancing and controlling the light emission of quantum emitters coupled to optically resonant nanostructures is essential for new light sources at the nanoscale, and for enhanced spectroscopies and biosensing^1^. The photoluminescence (PL) intensity of a quantum emitter at a position **r** coupled to a nanoantenna is influenced by three parameters, as expressed in the following equation:^2,3^

1 $$I\left({\bf{r}},{\omega }_{{exc}},{\omega }_{{em}}\right)={P}_{{exc}}\left({\bf{r}},{\omega }_{{exc}},{\omega }_{{em}}\right)\times {\phi }_{{em}}\left({\bf{r}},{\omega }_{{em}}\right)\times {C}_{{coll}}\left({\bf{r}},{\omega }_{{em}}\right)$$

where *P*_*exc*_(**r,***ω*_*exc*_) is the excitation rate, proportional to the excitation pump, *ϕ*_*em*_(**r,***ω*_*em*_) is the quantum yield related to the local density of photon states (LDOS) at the emission angular frequency *ω*_*em*_, and *C*_*coll*_(**r,***ω*_*em*_) is the collection efficiency, that depends on both the emission directivity and detection geometry.

Many works have been devoted to LDOS engineering for emission rate control^4–7^ and/or to dipole positioning for emission directivity control^8–11^. On the other hand, the influence of the pump has been rarely considered experimentally. In fact, the PL signal of a quantum emitter is either proportional to the LDOS or to the near-field intensity when the excited state is saturated or not, respectively^12–14^. In this article, we investigate in the nonsaturated regime the influence of focused cylindrical vector beams on the PL of a rare earth ion-doped thin film deposited on high refractive index dielectric nanostructures.

Rare-earth ion-based quantum emitters have been widely studied over the last decade because they are photostable and present narrow electronic transitions, corresponding to efficient electric (ED) or magnetic (MD) dipole transitions, either at the absorption^15^ or at the emission^16,17^. They have thus been used to probe the electric and magnetic components of light by placing them in the vicinity of plasmonic or dielectric antennas, where the electric and magnetic LDOS (E-LDOS and M-LDOS) can be adjusted and spatially separated^13,17–19^. To some extent, enhancing electric or magnetic Purcell effect could lead to applications in telecommunication using near-infrared emitters such as Er^3+^ ^20^, or efficient visible light sources and lamp phosphors using Eu^3+^ or Tb^3+^ ^21^. The MD transitions, interacting with the magnetic components of light^15,21,22^, may be even more interesting when they are coupled to high index dielectric nanoantennas, as in such nanostructures the magnetic field is known to be strongly enhanced at the magnetic dipole or quadrupole resonance wavelength^23–26^. Experimentally, a larger enhancement of the MD emitting transition compared to the ED emitting transition has been reported^13,27^. Theoretically, a few recent works predict a very strong enhancement of the magnetic Purcell effect, larger than 10^3^, in case of nanostructure design and M-LDOS optimization^7,28^.

Nevertheless, in addition to the nanoantenna design for tailoring the LDOS and the directional behavior as expressed in Eq. 1, the excitation beam is the third parameter that can be used to both spatially and spectrally shape the intensity of any optically driven light source. Focused cylindrical vector beams (CVB) of radial or azimuthal polarization can be very helpful as the electric and magnetic fields are spatially separated^15,29^, and can be used to selectively excite electric or magnetic resonance modes in dielectric nanoantennas^30,31^, as well as electric or magnetic dipole transitions in rare-earth ions^15^.

We thus show in the following that focused CVB can be used to spatially engineer the PL intensity of Eu^3+^-doped thin films deposited on Si nanorings, due to different controlled near-field hot spots.

## Results

**Fig. 1:**
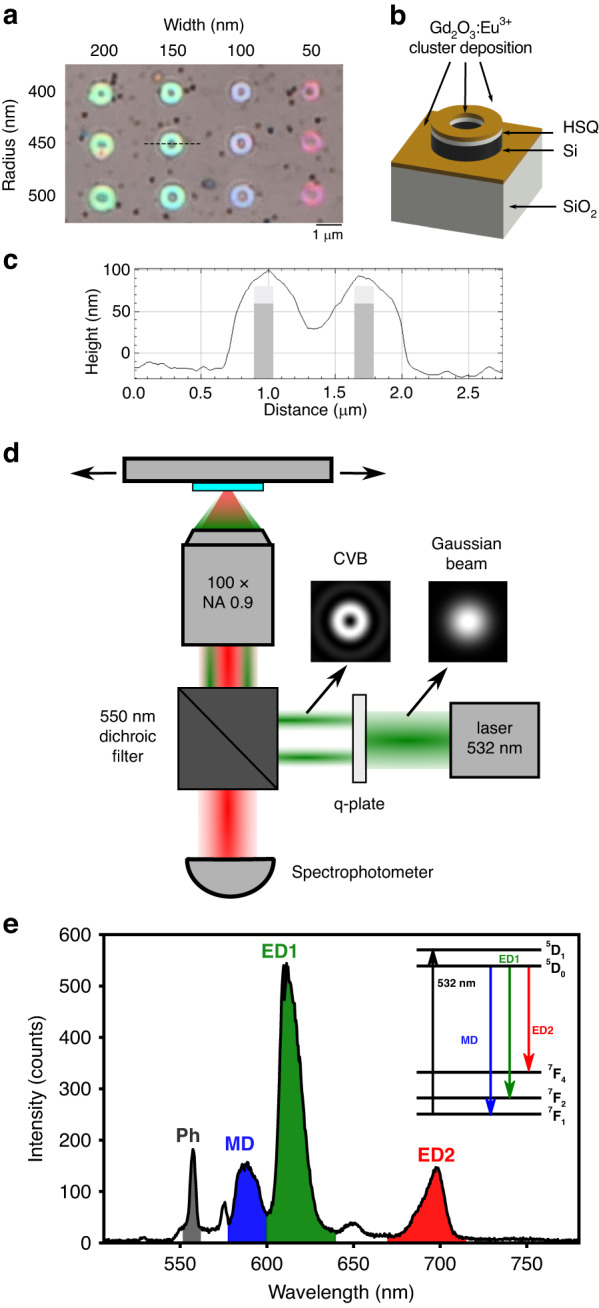
Experimental set-up and sample description. **a** Optical image of Si nanorings on SiO_2_ substrate. **b** Sketch of the sample showing the different layers. **c** AFM profile of a 400 nm radius Si nanoring after Eu^3+^:Gd_2_O_3_ cluster deposition corresponding to the dashed line in (**a**). **d** Sketch of the experimental set-up for PL imaging. **e** Typical spectrum taken on a Si-NR showing the Raman (gray band labeled Ph for phonons) and PL signals; inset: sketch in energy of the transitions in Eu^3+^ ions

In Fig. 1a, we show a bright field image of silicon nanoresonators covered by a 20 nm-thick film of Eu^3+^: Gd_2_O_3_ clusters obtained by low energy cluster beam deposition (LECBD)^13^. The Si nanorings (Si-NRs) are characterized by their outer radius *R* and width *W*. The film homogeneity is very good as shown by atomic force microscope (AFM) profile (see Fig. 1c).

The photoluminescence (PL) mappings are obtained by raster scanning the sample under a tightly focused laser beam. The excitation wavelength of 532 nm is resonant with the ^7^F_1_ → ^5^D_1_ electric dipole (ED) transition in Eu^3+^, and with a resonance mode of the Si-NRs (see supplemental information, Fig. S1)^15^. A typical PL spectrum is displayed in Fig. 1e. We focus on three main features corresponding to ^5^D_0_ → ^7^F_1_ centered around 590 nm (MD transition), ^5^D_0_ → ^7^F_2_ around 610 nm (ED1 transition), and ^5^D_0_ → ^7^F_4_ around 690 nm (ED2 transition)^15,17^. Notice that, when scanning the Si-NRs, the Raman spectrum due to two optical phonon processes can be detected (gray band labelled Ph in the spectrum of Fig. 1e). This is helpful defining accurately the location of the Si nanoantennas.

**Fig. 2:**
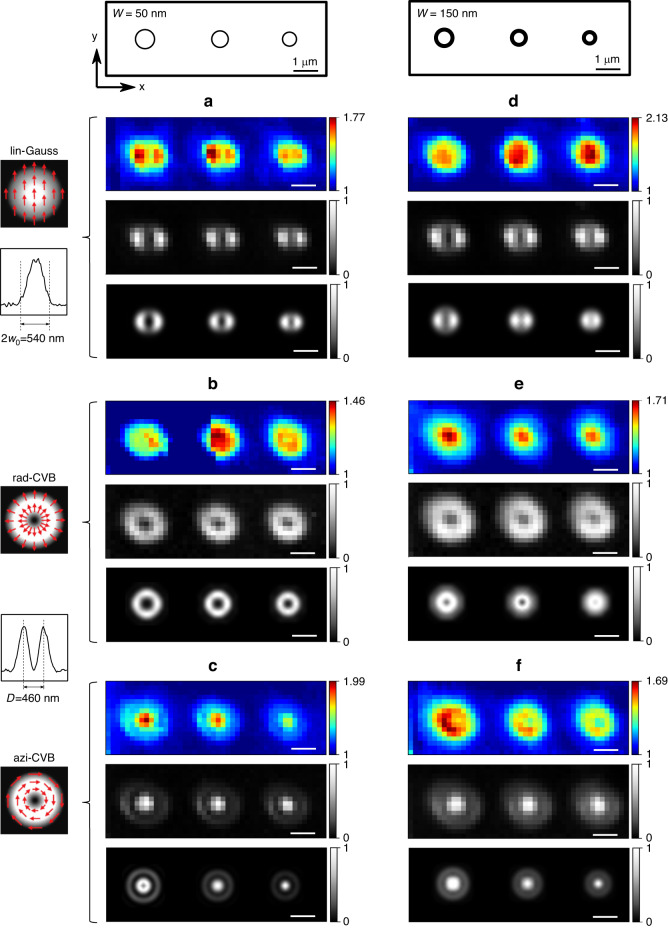
Photoluminescence and Raman mappings. Left column: Si-NRs of *W* = 50 nm width (radius *R* = 500, 450, and 400 nm from left to right) excited by, from top to bottom, (**a**) lin-Gauss, (**b**) rad-CVB and (**c**) azi-CVB. For each excitation are shown the PL mapping (top row), the Raman scattering mapping (middle row), and the calculated electric field intensity distribution inside the Si-NR. The PL maps are normalized to the signal far from the Si-NRs. Both experimental Raman and calculated electric field intensity maps are normalized to local maximum (no Raman signal outside the Si-NRs). Right column: same as left column for Si-NRs of *W* = 150 nm width, excited by (**d**) lin-Gauss, (**e**) rad-CVB and (**f**) azi-CVB. The schemes showing the different nanorings is given on top of the mappings described above. Left to the PL maps are reported the different polarizations and the experimental intensity profiles in the focal plane of the Gaussian beam (*w*_0_ is the waist at 1*/e*^2^), and of both CVB (*D* is the diameter of the doughnut-shaped beam)

In the following, we address the Si Raman signal and the total PL intensity given by the integrated intensity of the three contributions (MD, ED1, and ED2). Typical PL and Raman scattering (RS) mappings for different polarizations and Si-NRs dimensions are presented in Fig. 2. The linear polarization of the Gaussian excitation beam is referred to as lin-CVB. The doughnut beam shape associated to azimuthal (resp. radial polarization) is referred to as azi-CVB (resp. rad-CVB). In all cases, the power density on the sample was kept low enough to stay below the saturation regime of the excited state (see supplementary information, Fig. S2), thus avoiding the PL being governed by the LDOS only. In the low power regime, as previously mentioned, we expect the PL to be dependent mainly on the near-field at the excitation wavelength^12–14^.

The main results that can be underlined are: (i) the RS maps are dependent on the excitation polarization only, and (ii) the PL maps not only depend on the excitation polarization but also on the Si-NR width *W*.

### Raman mappings of Si nanorings

As shown in Fig. 2, the Raman mappings give the same signature for all nanorings for a given laser polarization. There are two lobes for the (vertical) linear polarization, a doughnut shape for the radial polarization, and a centered spot for the azimuthal polarization. The fact that the signal intensity map depends only on the laser polarization for a nanoring shape leads us to the assumption that the Raman signal is driven by the local electric field inside the nanoring^32,33^. To prove this, we calculated by FDTD (See Materials and Methods) the electric-field intensity inside the Si-NR in each point of the raster scan of the laser beam. The simulations are also displayed in Fig. 2 below the experimental RS maps.

Both the experimental Raman maps and the calculated internal field intensity maps are in perfect agreement, validating our hypothesis.

### Photoluminescence mappings of Eu^3+^:Gd_2_O_3_ deposited films on nanorings

Confirming AFM images and previous experiments^13^, the cluster-deposited film is highly homogeneous as shown by the constant intensity observed in PL mappings far from the Si nanostructures.

There is a slight enhancement of the Eu^3+^ emission intensity above the Si-NR, but more important we point out a strong modification of the spatial distribution of the PL intensity as function of the excitation polarization. This spatial distribution is also strongly dependent on the nanoring dimensions in the case of the rad-CVB and azi-CVB excitations (see Fig. 2).

For linear polarized Gaussian beam, the PL enhancement evolves from two lobes for *W* = 50 nm to a large central maximum with increasing width *W*. For radial polarization, the PL enhancement evolves from a doughnut shape for *W* = 50 nm to a narrow dotted maximum in the ring center with increasing width *W*. This behavior is fully reversed in the case of azimuthal polarization, with an evolution from narrow dotted maximum for *W* = 50 nm to the doughnut shape with increasing width *W*.

## Discussion

To understand the experimental results, we calculated using FDTD (See Materials and Methods) the electric near-field intensity distribution by raster scanning the different CVBs inside the Eu^3+^-doped film (i.e., following a profile at 10 nm above the substrate surface and the Si-NR top surface including the HSQ layer). The near-field intensity distribution is calculated for each laser spot center position of the raster scan. The raster scan and the construction point by point of the near-field map are illustrated in a movie available in supplementary information (section F).

**Fig. 3:**
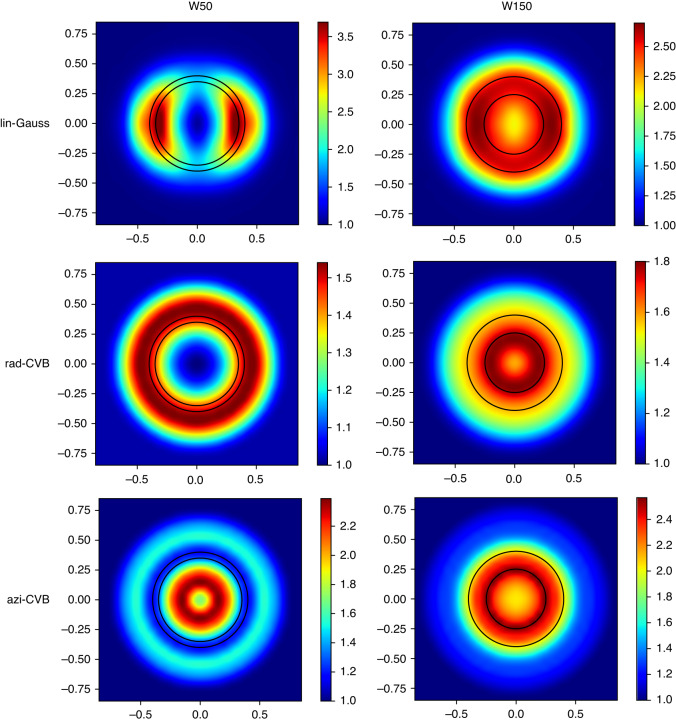
FDTD simulations of normalized near-field electric intensity distribution in the Eu^3+^-doped layer. From top to bottom, normalized near-field electric intensity distribution obtained by raster scanning the lin-Gauss, rad-CVB and azi-CVB excitation, respectively. The position in height of the raster scan is along the Eu^3+^:Gd_2_O_3_ layer (10 nm above the silica substrate or the HSQ layer covering the 400 nm radius Si-NR). Left columns: Si-NRs of *W* = 50 nm. Right columns: Si-NRs of *W* = 150 nm (The nanoring contour is plotted as black line)

The results are given in Fig. 3 in the case of 400 nm radius Si-NRs with two selected widths *W* = 50 nm and *W* = 150 nm. There is a qualitative agreement between the near-field simulations of Fig. 3 and the PL experiments. Indeed, in the case of the azi-CVB (bottom line of Figs. 2 and 3), there is an obvious tendency for the near-field intensity maximum to form a doughnut above the Si-NR contour for the Si-NR with the largest width *W* = 150 nm, to a reduced spot more centered inside the Si-NR contour for the narrowest *W* = 50 nm. The behavior is reversed in the rad-CVB case (middle line of Figs. 2 and 3): large doughnut shape for *W* = 50 nm and centered spot for *W* = 150 nm. In the lin-CVB case, we observe an evolution from the two lobes for the narrowest *W* to a larger central spot for the largest *W* as in the PL maps.

As we chose the laser power to stay below the saturation regime (where the emission is driven by the LDOS)^12,14^, and that the excitation wavelength at 532 nm corresponds to an electric dipole transition^15^, the good agreement with the electric near-field simulations shows that the PL is governed by the electric near-field distribution around and above the Si nanostructures. Our results also prove that the PL response can be tuned by the combination of Si nanoantennas and excitation beam shape and polarization.

Depending on these parameters, it is thus possible to have a narrow-spotted PL maximum, which can be seen in both cases of azi-CVB and *W* = 50 nm diameter Si-NR (Fig. 2c), and rad-CVB and *W* = 150 nm diameter Si-NR (Fig. 2e). By increasing the objective *NA* or the local electric field intensity (for instance by decreasing both the nanoring and central hole diameters), we could expect an enhanced PL in a reduced spot size, improving considerably the spatial resolution. We point out that this effect could be even higher using the magnetic field with the appropriate excitation wavelength (527.5 nm) resonant with the ^7^F_0_ → ^5^D_1_ magnetic dipole transition in Eu^3+^ ^15^, as the magnetic field intensity is usually much larger than the electric field inside dielectric nanostructures^25,34^. There is however an experimental challenge of filling a hole of about 50 nm in diameter by the emitters.

### Branching ratios and influence of radiative LDOS and light collection

After having investigated how the total PL intensity could be controlled by the excitation beam, we now focus on the influence of the system on the different competing emission channels, referred to as MD, ED1 and ED2 centered around 590 nm, 610 nm and 700 nm, respectively (See the experimental set-up section and Fig. 1). The usual parameters used to describe how the emission channels are redistributed are the branching ratios, given by:

2 $${\beta }_{i}={I}_{i}/{I}_{{tot}}={\Gamma }_{i}^{{rad}}/{\Gamma }_{{tot}}^{{rad}}$$

where *I*_*i*_ is the integrated intensity of the transition labeled *i* (MD, ED1, or ED2), *I*_*tot*_ is the sum of the three contributions, ${\Gamma }_{i}^{{rad}}$ is the radiative emission rate of the transition labeled *i*, and ${\Gamma }_{{tot}}^{{rad}}$ is the sum of the radiative emission rates of each transition. This method allows to connect the intensity measured in stationary PL experiments to the radiative electric and magnetic LDOS^5,19,35^.

**Fig. 4:**
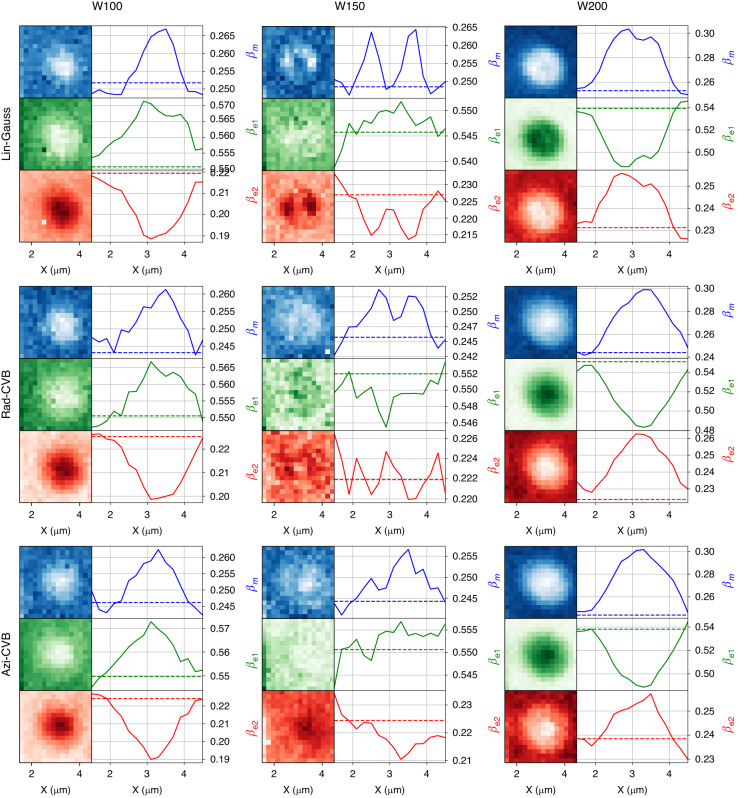
Branching ratios. Branching ratio profiles for the different transitions so called MD (*β*_*m*_, blue), ED1 (*β*_*e*1_, green) and ED2 (*β*_*e*2_, red). The 1D profiles are taken along a horizontal line of the 2D mappings. The dashed lines in the spectra correspond to the BR of Eu^3+^-doped film without any Si-NR antenna influence, referred to as BR_*ref*_. The positions where the BRs coincide with their respective BR_*ref*_ values correspond to positions outside Si-Nrs

We show in Fig. 4 the BR maps and 1D profiles for a Si-NR of 400 nm radius (additional maps and profiles for several radii and widths are given in supplementary information in Fig. S3 and Fig. S4). Though the influence of the excitation beam polarization is still visible, we point out the additional influence of the radiative LDOS as the BRs are affected as function of the Si-NR width. For the smallest *W* (100 nm and below, left column of Fig. 4), the MD and ED1 emitting transitions tend to be favored to the detriment of ED2 transition. For the largest *W* = 200 nm width, the MD is still enhanced while ED1 is now decreased to the benefit of ED2 (right column of Fig. 4). It seems that the *W* = 150 nm case corresponds to the transition between the extreme behaviors described above. Notice that the same behavior is found for different Si-NR radii (500–350 nm range) for a given *W* value. The global behavior is also reproducible on different samples. Outside the Si-NRs, the BRs of each transition tend toward their respective limit values in a homogeneous environment. Such values referred to as BR_*ref*_ (see Fig. 4 and Fig. S4 of supplemental information) are close to those of other works^17,19^.

The calculated magnetic and electric LDOS (see Fig. S5 in supplementary information) do not fit the experiments, as the BRs depend only on the part of the radiative decay that enters the microscope objective^19^. According to Eq. 1, more complex simulations are needed taking into account the LDOS through the quantum yield *ϕ*_*em*_(**r,***ω*_*em*_), and the emission directivity and detection geometry *C*_*coll*_(**r,***ω*_*em*_).

We thus show that it is possible to shape the PL spatially, favoring different hot spots as function of the excitation beam polarization, while either enhancing or quenching a specific emission line of the Eu^3+^-doped film.

In conclusion, we investigated the photoluminescence mappings of Eu^3+^-doped cluster-deposited films on Si nanorings using different cylindrical vector beams. In addition to the photon LDOS, we show that the excitation beam shape and polarization have an important effect. Radial and azimuthal polarized beams exciting ring shape nanoantennas allow to tune the near-field hot spots, hence the local PL enhancement.

Our results show that it could be possible to spatially shape the photoluminescence of quantum emitters coupled to dielectric nanoantennas by both the excitation and emission channels. More complex dielectric nanoantennas need to be designed to be resonant at an absorption transition excited by a chosen cylindrical vector beam, and at a specific emitting transition while quenching others. Rare earth ions supporting magnetic transitions at both absorption and emission are very good candidates to benefit from a very high local magnetic field enhancement inside resonant dielectric nanoantennas at the excitation wavelength, combined to magnetic and electric LDOS engineering to control the different emission channels.

## Materials and methods

Si nanostructures are fabricated by electron beam lithography followed by reactive ion etching in a single crystal Si layer transferred on a fused silica (SiO_2_) substrate, referred to as silicon on silica (SOS). The Si-NR height is fixed by the Si top layer of the SOS (*H* = 90 nm). We chose nanorings (NRs) for their doughnut-like shape similar to the CVB symmetry.

The distance between the Si-NR top surface and the Eu^3+^: Gd_2_O_3_ deposit is about 20 nm, corresponding to the thickness of the hydrogen silsesquioxane (HSQ) resist, which was left on top of the Si-NRs during the fabrication process to protect the smallest structures from etching or lift-off (see Fig. 1b). The optical constants of the HSQ resist are the same as those of silica.

The experimental set-up shown in Fig. 1d, and the data acquisition software are homemade^36^. We used a confocal microscope (Nikon Ti2) to focus the laser beam through an objective of numerical aperture *NA* = 0.9. The linear polarization of the Gaussian excitation beam can be modified by a half-wave plate. The doughnut beam shape associated to azimuthal or radial polarization is obtained by adding a q-plate after the half-wave plate and before the microscope. We verified that the chosen polarization is conserved after reflection on the dichroic mirror (Fig. 1d). The spectra are recorded in each point of the raster scan using a spectrometer (Andor Shamrock 193, grating of 300 groves.mm^−1^) and a CCD detector (Andor Idus).

For electro-dynamical simulations, we used the Meep software package based on the finite-difference time-domain (FDTD) method^37^, along with python script packages that we developed for implementation of CVB excitations in Meep^31^.

### Supplementary information

Supplementary information

Raster scan movie

## References

[1] Krasnok A (2018). Spectroscopy and biosensing with optically resonant dielectric nanostructures. Adv. Optical Mater..

[2] Koenderink AF (2017). Single-photon nanoantennas. ACS Photon..

[3] Bidault S, Mivelle M, Bonod N (2019). Dielectric nanoantennas to manipulate solid-state light emission. J. Appl. Phys..

[4] Li JQ, Verellen N, Van Dorpe P (2017). Enhancing magnetic dipole emission by a nano-doughnut-shaped silicon disk. ACS Photon..

[5] Sanz-Paz M (2018). Enhancing magnetic light emission with all-dielectric optical nanoantennas. Nano Lett..

[6] Mignuzzi S (2019). Nanoscale design of the local density of optical states. Nano Lett..

[7] Brûlé Y (2022). Magnetic and electric Purcell factor control through geometry optimization of high index dielectric nanostructures. Opt. Express.

[8] Curto AG (2010). Unidirectional emission of a quantum dot coupled to a nanoantenna. Science.

[9] Poumirol JM (2020). Unveiling the optical emission channels of monolayer semiconductors coupled to silicon nanoantennas. ACS Photon..

[10] Wiecha PR (2019). Design of plasmonic directional antennas via evolutionary optimization. Opt. Express.

[11] Humbert M (2023). Large-scale controlled coupling of single-photon emitters to high-index dielectric nanoantennas by AFM nanoxerography. Nanoscale.

[12] Girard C (2005). Generalized Bloch equations for optical interactions in confined geometries. Chem. Phys. Lett..

[13] Wiecha PR (2019). Enhancement of electric and magnetic dipole transition of rare-earth-doped thin films tailored by high-index dielectric nanostructures. Appl. Opt..

[14] Majorel C (2020). Quantum theory of near-field optical imaging with rare-earth atomic clusters. J. Opt. Soc. Am. B.

[15] Kasperczyk M (2015). Excitation of magnetic dipole transitions at optical frequencies. Phys. Rev. Lett..

[16] Karaveli S, Zia R (2011). Spectral tuning by selective enhancement of electric and magnetic dipole emission. Phys. Rev. Lett..

[17] Aigouy L (2014). Mapping and quantifying electric and magnetic dipole luminescence at the nanoscale. Phys. Rev. Lett..

[18] Mivelle M (2015). Strong modification of magnetic dipole emission through diabolo nanoantennas. ACS Photon..

[19] Rabouw FT, Prins PT, Norris DJ (2016). Europium-doped NaYF_4_ nanocrystals as probes for the electric and magnetic local density of optical states throughout the visible spectral range. Nano Lett..

[20] Kalinic B (2020). All-dielectric silicon nanoslots for Er^3+^ photoluminescence enhancement. Phys. Rev. Appl..

[21] Baranov DG (2017). Modifying magnetic dipole spontaneous emission with nanophotonic structures. Laser Photon. Rev..

[22] Wiecha PR (2018). Decay rate of magnetic dipoles near nonmagnetic nanostructures. Phys. Rev. B.

[23] Rolly B (2012). Promoting magnetic dipolar transition in trivalent lanthanide ions with lossless Mie resonances. Phys. Rev. B.

[24] Albella P (2013). Low-loss electric and magnetic field-enhanced spectroscopy with subwavelength silicon dimers. J. Phys. Chem. C..

[25] Bakker RM (2015). Magnetic and electric hotspots with silicon nanodimers. Nano Lett..

[26] Matsumori A, Sugimoto H, Fujii M (2022). Silicon nanosphere with accessible magnetic hotspot. Adv. Opt. Mater..

[27] Sugimoto H, Fujii M (2021). Magnetic Purcell enhancement by magnetic quadrupole resonance of dielectric nanosphere antenna. ACS Photon..

[28] Rocco D (2020). Giant electric and magnetic Purcell factor in dielectric oligomers. J. Opt. Soc. Am. B.

[29] Dorn R, Quabis S, Leuchs G (2003). Sharper focus for a radially polarized light beam. Phys. Rev. Lett..

[30] Woźniak P, Banzer P, Leuchs G (2015). Selective switching of individual multipole resonances in single dielectric nanoparticles. Laser Photon. Rev..

[31] Montagnac M (2022). Engineered near- and far-field optical response of dielectric nanostructures using focused cylindrical vector beams. J. Appl. Phys..

[32] Dmitriev PA (2016). Resonant Raman scattering from silicon nanoparticles enhanced by magnetic response. Nanoscale.

[33] Raza S, Kristensen A (2021). Raman scattering in high-refractive-index nanostructures. Nanophotonics.

[34] Yang ZJ, Zhao Q, He J (2017). Boosting magnetic field enhancement with radiative couplings of magnetic modes in dielectric nanostructures. Opt. Express.

[35] Chacon R (2022). Vectorial probing of electric and magnetic transitions in variable optical environments and vice-versa. Nanotechnology.

[36] Weber SJ (2021). PyMoDAQ: an open-source python-based software for modular data acquisition. Rev. Sci. Instrum..

[37] Oskooi AF (2010). MEEP: a flexible free-software package for electromagnetic simulations by the FDTD method. Comput. Phys. Commun..

