# Supplementary material for: Control of light emission of quantum emitters coupled to silicon nanoantenna using cylindrical vector beams
Source: Light Sci Appl. 2023 Sep 19;12:239. doi: 10.1038/s41377-023-01229-9 (PMC10509260; doi:10.1038/s41377-023-01229-9)
Supplement: Supplementary file 1 — Supplementary information [file 41377_2023_1229_MOESM1_ESM.docx]

*Supplementary information for*:

Control of light emission of quantum emitters coupled to silicon nanoantennas using cylindrical vector beams

# Martin Montagnac^1^, Yoann Bruˆl´e^2^, Aur´elien Cuche^1^, Jean-Marie Poumirol^1^, S´ebastien J. Weber^1^, Jonas Mu¨ller^3^, Guilhem Larrieu^3^, Vincent Larrey^4^, Franck Fournel^4^, Olivier Boisron^5^, Bruno Masenelli^6^, G´erard Colas des Francs^2^, Gonzague Agez^1^,[^∗^](#_bookmark0) and Vincent Paillard^1^[^†^](#_bookmark1)

# ^1^*CEMES-CNRS, Universit´e de Toulouse, Toulouse, France*

^2^*ICB, Universit´e de Bourgogne, CNRS, Dijon, France*

^3^*LAAS-CNRS, Universit´e de Toulouse, Toulouse, France*

^4^*CEA-LETI, Universit´e Grenoble-Alpes, Grenoble, France*

# ^5^*Universit´e de Lyon, Universit´e Lyon 1, CNRS UMR 5510, ILM, Villeurbanne, France*

^6^*Universit´e de Lyon, INSA Lyon, CNRS, Ecole Centrale de Lyon, Universit´e Lyon 1, CPE, UMR 5270, INL, Villeurbanne, France*

## CONTENTS

1. [Resonances of Si nanorings](#_bookmark2) 2
2. [Power Dependence of PL intensity](#_bookmark3) 3
3. [Branching ratio mappings](#_bookmark4) 4
4. [Branching ratio profiles](#_bookmark6) 5
5. [Local Density of States](#_bookmark7) 6
6. [Raster scan movie: point by point construction of near-field map](#_bookmark8) 7

^∗^ [gonzague.agez@cemes.fr](mailto:gonzague.agez@cemes.fr)

^†^ [vincent.paillard@cemes.fr](mailto:vincent.paillard@cemes.fr)

## Resonances of Si nanorings


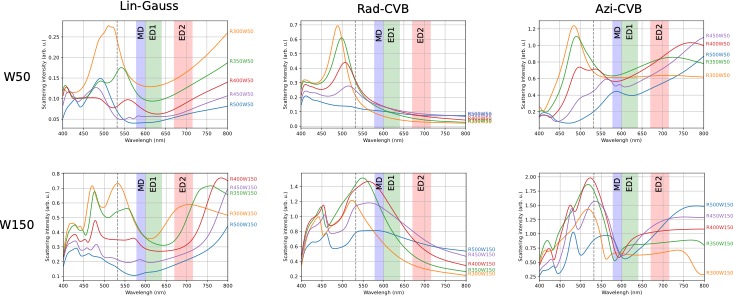


FIG. S1 FDTD simulations of scattering spectra of Si nanorings of different radii and widths excited by lin-Gaus, rad-CVB and azi-CVB. The excitation beam is centered on the Si-NR. The Si-NRs are roughly resonant with the excitation the 532 nm excitation (vertical dashed line in the spectra). The blue, green and red bands correspond to the ^5^D_0_→^7^F_1_ around 590 nm (MD transition), ^5^D_0_→^7^F_2_ around 610 nm (ED1 transition), and ^5^D_0_→^7^F_4_ around 690 nm (ED2 transition), respectively.

## Power Dependence of PL intensity

FIG. S2 Photoluminescence intensity recorded on a Si-NR. The dashed line shows the power used for the experiments, below the PL saturation.

## Branching ratio mappings

FIG. S3 Branching ratio mappings for several nanoring radii (500, 450 and 400 nm) and widths, in complement of Figure 4 of the main text.

## Branching ratio profiles

**
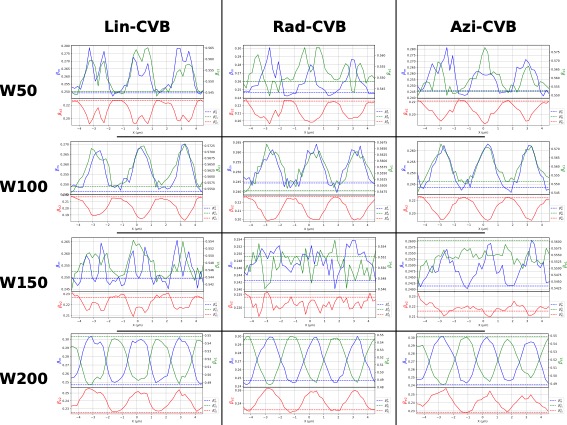
**

FIG. S4 Branching ratio 1D profiles for several nanorings corresponding to the mappings in Figure [S3](#_bookmark5).

## Local Density Of States

FIG. S5 Calculated magnetic and electric Purcell factor mapping for nanorings with radius of 400 nm and increasing widths from top to bottom.

- 1. **
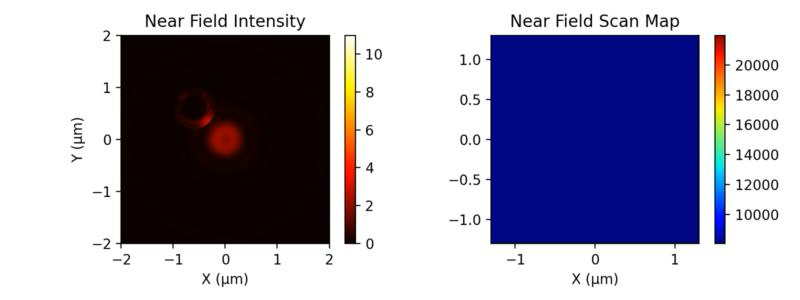
Raster scan movie: point by point construction of near-field map**

FIG. S6 The movie RasterScan.avi shows the fixed laser spot raster scanned by a nanoring (left display), and the corresponding point by point construction of the near-field map intensity (right display). Below is a snapshot taken from the movie, this movie can be downloaded in supplementary information.
